# The Current Status of the Pharmaceutical Potential of *Juniperus* L. Metabolites

**DOI:** 10.3390/medicines5030081

**Published:** 2018-07-31

**Authors:** Wilson R. Tavares, Ana M. L. Seca

**Affiliations:** 1 Faculty of Sciences and Technology, University of Azores, 9501-801 Ponta Delgada, Portugal; wrt-94@hotmail.com; 2 Department of Chemistry & QOPNA-Organic Chemistry, Natural Products and Food Stuffs, University of Aveiro, Campus de Santiago, 3810-193 Aveiro, Portugal; 3 cE3c—Centre for Ecology, Evolution and Environmental Changes/Azorean Biodiversity Group & Faculty of Sciences and Technology, University of Azores, Rua Mãe de Deus, 9501-321 Ponta Delgada, Portugal

**Keywords:** *Juniperus*, secondary metabolites, diterpenes, flavonoids, lignans, cytotoxic, antitumor, antibacterial, amentoflavone, deoxypodophyllotoxin

## Abstract

**Background:** Plants and their derived natural compounds possess various biological and therapeutic properties, which turns them into an increasing topic of interest and research. *Juniperus* genus is diverse in species, with several traditional medicines reported, and rich in natural compounds with potential for development of new drugs. **Methods:** The research for this review were based in the Scopus and Web of Science databases using terms combining *Juniperus*, secondary metabolites names, and biological activities. This is not an exhaustive review of *Juniperus* compounds with biological activities, but rather a critical selection taking into account the following criteria: (i) studies involving the most recent methodologies for quantitative evaluation of biological activities; and (ii) the compounds with the highest number of studies published in the last four years. **Results:** From *Juniperus* species, several diterpenes, flavonoids, and one lignan were emphasized taking into account their level of activity against several targets. Antitumor activity is by far the most studied, being followed by antibacterial and antiviral activities. Deoxypodophyllotoxin and one dehydroabietic acid derivative appears to be the most promising lead compounds. **Conclusions:** This review demonstrates the *Juniperus* species value as a source of secondary metabolites with relevant pharmaceutical potential.

## 1. Introduction

Plants have been used by humans since the start of mankind thousands of years ago as construction material [1], clothing [2], and obviously, as food and drugs [3]. Although scientific knowledge has permitted the development of medicine to today’s standards based on herbal and traditional medicines, the oldest form of medicine known to man, they are still used around the world [4]. The use of plants themselves, their derived natural compounds and their biological and therapeutic properties have become a topic of increasing interest and investigation not only in modern medicine and pharmacology [5], but also in food and cosmetics industries [6].

*Juniperus* species are a good bet in the development of new drugs with natural compounds, since it is a diverse genus (75 species of *Juniperus* [7]) with several traditional medicinal applications reported. For example, *Juniperus excelsa* M.Bieb. is used to treat abdominal spasm, asthma, diarrhea, fever, gonorrhea, headache, and is also useful as antihypertensive, diuretic, carminative, appetizer, anticonvulsant, and flavoring agent [8]. In Turkey, powdered *Juniperus oxycedrus* subsp. *oxycedrus* L. berries are consumed to lower blood glucose levels [9], while in Mexico, *Juniperus communis* L. is used to treat respiratory problems, gastrointestinal infections, cardiovascular and/or blood disorders, and as astringent [10]. Use of *J. communis* also covers the treatment of urinary problems, migraines, diabetes, gonorrhea, and skin irritations [11].

The extracts and secondary metabolites from *Juniperus* species exhibit also interesting bioactivities [12,13], especially the *Juniperus oxycedrus* L. and *J. communis,* two of the most studied species in terms of their phytochemistry, pharmacological, and therapeutic effects [14,15]. It can be highlighted that extracts and compounds from both plants exhibit antimicrobial, antioxidant, antidiabetic, anti-inflammatory, anticonvulsant, analgesic, and cytotoxic activities [14,15], and additionally *J. communis* also possess antifertility, hepatoprotective, diuretic, neuroprotective, antiparasitic, and anti-ulcer properties [14].

Some of the most relevant studies published in recent years on the bioactivities of *Juniperus* extracts show clearly that *Juniperus* continues to be a hot spot in research on natural products as well as contribute to further highlight the pharmacological potential of this genus and of its chemical constituents. For example, Jung et al. [16] reports the butyrylcholinesterase (BChE) inhibitory activity of the compound valenc-1(10), 3(4), 11(12)-trien-2-one isolated from *Juniperus chinensis* L. with an IC_50_ value of 68.45 μM (IC_50_ = 18.75 μM for berberine). In Lee and colleagues work [17], *Juniperus rigida* Siebold & Zucc. fruit ethanol extract was showed to possess anti-atopic properties in in vivo oxazolone- and 2,4-dinitrochlorobenzene(DNCB), and induced atopic dermatitis in mice models. It was suggested that the therapeutic effect verified by this extract occurs by decreasing the overproduction of interleukin 4 (IL-4) and immunoglobulin E (IgE) and accelerating skin barrier recovery function. Groshi et al. [18] assessed the cytotoxicity of the polar extract (methanol), and non-polar extracts (dichloromethane and *n*-hexane) of *Juniperus phoenicea* L. leaves against four human cancer cell lines concluding that the dichloromethane extract was the most cytotoxic extract against the lung carcinoma cell line A549 (IC_50_ = 13 μg/mL), while *n*-hexane extract exhibits the broadest spectrum of activity with IC_50_ values of 10, 14, 16, and 40 μg/mL against hepatocellular carcinoma cell line HepG2, human breast cancer cell line MCF-7, human lung carcinoma A549, and human bladder carcinoma cell line EJ138, respectively. On the other hand, imbricataloic acid isolated from *Juniperus phoenicea* var. *turbinata* (Guss.) Parl. (syn. *Juniperus turbinata* Guss.) ethanol extract showed the strongest cytotoxic activity (IC_50_ values of 0.06, 0.114, and 0.201 μM on human colon cancer HCT116, human malignant melanoma A375, and human breast adenocarcinoma MDA-MB-231 cell lines, respectively), being several times more potent than the reference compound cisplatin (IC_50_ values of 1.87 to 11.86 μM) [19] indicating that imbricataloic acid make a promising anticancer drug candidate.

The essential oil of *Juniperus* species are also a research target once they exhibit a great diversity of bioactivities. For example, the essential oil of *J. phoenicea* var. *turbinata* (syn. *Juniperus turbinata* Guss.) exhibits cytotoxic effects against HCT116, A375, and MDA-MB-231 human tumor cell lines, in a concentration-dependent inhibitory effect with IC_50_ values of 9.48–33.69 μg/mL [19]. *Juniperus oxycedrus* essential oil exhibited high antitrypanosomal activity (IC_50_ of 0.9 μg/mL) against *Trypanosoma brucei brucei*, with no cytotoxic effects on RAW 267.4 macrophage cell line showing the highest selectivity index (63.4) [20]. The authors of this study suggest that α-pinene would likely be the responsible for the *J. oxycedrus* essential oil antitrypanosomal properties. In another work using male mice [21], *Juniperus virginiana* L. essential oil at 400 and 800 mg/kg showed anxiolytic effect, although it failed to inhibit the anxiety-related behavior by light-dark box.

The interest of the *Juniperus* species is also at the nutritional/functional food level and some studies, including in vivo studies on this subject, have recently been published. Inci and colleagues [22] found that low supplementation levels of *J. communis* berry (0.5% and 1%) in Japanese quails (*Coturnix coturnix japonica*) diets have positive impacts on some body qualities, feed intake, and live weight. *Juniperus* species are also valuable in terms of their wood since it is a viable construction material classified as durable or even very durable, like *J. communis* case [23]. In this context, Ateş et al. [24] suggest that *Juniperus foetidissima* Willd. could be aimed for new natural wood preservatives development since its methanol extract reported antifungal activity against *Pleurotus ostreatus* with an IC_50_ value of 0.30 μg/μL.

Besides all the bioactive activities and other benefit effects of *Juniperus* species mentioned above, it should be noticed that toxicity side effects were found in *Juniperus* species such as the spoonful ingestion of *J. oxycedrus* extract of branches can cause poisoning, leading to fever, hepatotoxicity, renal failure, severe hypotension, and severe cutaneous burns on the face [25]. Adverse effects were also mentioned by Prinsloo and colleagues [3] to *Juniperus sabina* L. that contains thujone, a neurotoxic compound, and to *Juniperus scopulorum* Sarg., which contains safrole, a liver carcinogen substance [3].

Taking into account the abovementioned bioactivities of some *Juniperus* species, their importance as a source of novel natural compounds is well cleared. The increased interest and investigation of these species lead to new discoveries of interesting and promising metabolites. *Juniperus* L. metabolites pharmaceutical potential has been previously well reviewed in 2006 [12] and in 2015 [13], thus this work aims to update the information relative to the recently published studies involving *Juniperus* species secondary metabolites. It is important to highlight that this is not an exhaustive review of all the studies regarding compounds with biological activities form *Juniperus* species, but rather a selection taking into account the compounds whose biological activity and mechanism of action show that they are compounds with high pharmacological potential.

## 2. Bioactive Secondary Metabolites from *Juniperus* Species

### 2.1. Terpenoids

#### 2.1.1. Dehydroabietic Acid

**Figure 1:**
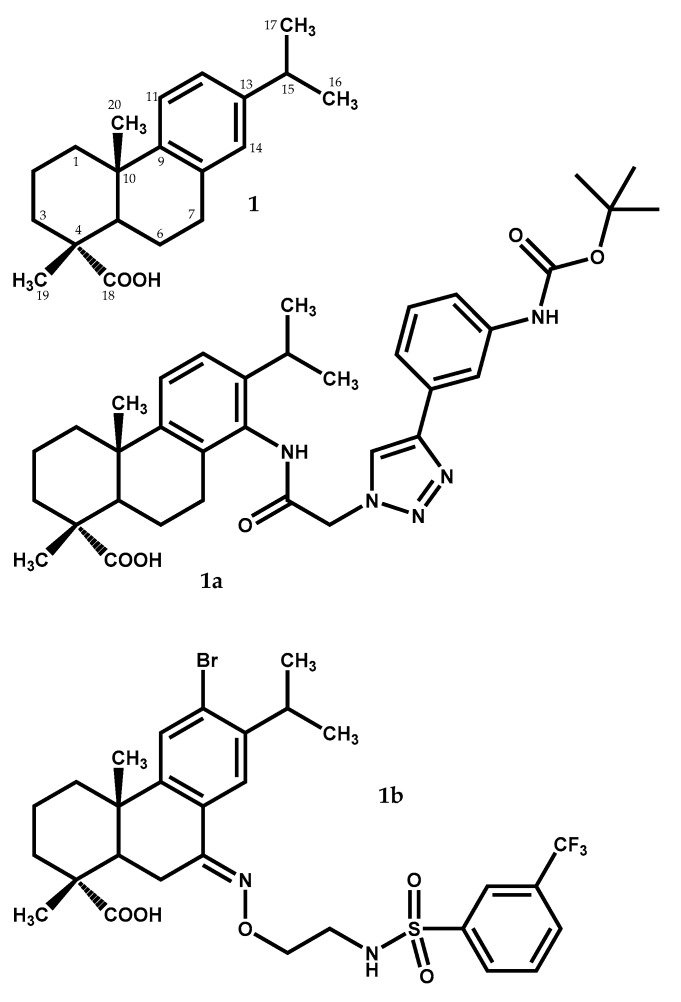
Dehydroabietic acid (**1**) identified in *Juniperus* species and its derivatives (**1a** and **1b**) with significant cytotoxic and antibacterial activities.

The dehydroabietic acid (1) (Figure 1) has a lipophilic abietan-8,11,13-trien structure with only one polar substituent, the equatorial carboxylic group at C-4. This acid is widely distributed in nature, being present in *J. oxycedrus*, *J. phoenicea*, and *Juniperus brevifolia* (Seub.) Antoine [12,13]. This compound has been considered as an interesting starting material for the synthesis of new compounds which means, an excellent leader compound, with important biological properties, having at least two hundred dehydroabietane derivatives described in literature [26]. Dehydroabietic acid (1) and its derivatives display not only antiviral [27] and antitumor [28,29] effects, but also gastroprotective [30], antimicrobial [31], and anti-inflammatory [32] properties. 

More recently, new interesting dehydroabietic acid derivatives were studied. Hou and colleagues [33] accessed the in vitro antiproliferative activity of various dehydroabietic acid derivatives possessing a 1,2,3-triazole-tethered nucleus at C-14, against four different human cancer cell lines, showing that the majority of the newly synthesized derivatives displayed effective antiproliferative activities, being the presence of 1,2,3-triazole moiety substituted on C-4 crucial to the high cytotoxic activity. The dehydroabietic acid methyl ester derivative (1a) (Figure 1), with the substituent (2-(4-(3-(*tert*-butoxycarbonylamino)phenyl)-1*H*-1,2,3-triazol-1-yl)acetamido) at C-14, was the most potent derivative tested, exhibiting better IC_50_ values (i.e., 0.7 to 1.2 μM) against the tested cells lines (PC-3, SK-OV-3, MDA-MB-231 and MCF-7 human cell lines) than the clinical anticancer drug fluorouracil (5-Fu) (5.2 to 24.5 μM IC_50_ values). Moreover, it also demonstrated weak cytotoxicity against HL-7702 and HFF-1 normal cells. These results imply that, with proper structure modifications, these types of derivatives could be aimed for development into a new anticancer natural product-like.

In a very recent work [34], the antibacterial activity of various N-sulfonaminoethyloxime derivatives of dehydroabietic acid was assessed against *Staphylococcus aureus* Newman strain and multidrug-resistant *Staphylococcus aureus* strains (NRS-1, NRS-70, NRS-100, NRS-108, and NRS-271). The results showed that these dehydroabietic acid derivatives showed great antibacterial effect with minimum inhibitory concentration (MIC) values ranging from 0.78 to 1.56 μg/mL against the strains tested. With a MIC of 0.39 to 0.78 μg/mL (MIC = 0.63–1.2 μM) against *Staphylococcus aureus* Newman, the meta-CF_3_ phenyl derivative (1b) (Figure 1) showed the highest antibacterial activity, similar to the positive-control compound vancomycin, that had a MIC of 0.78 to 1.56 μg/mL (MIC = 0.54–1.1 μM) against the same bacteria strain.

#### 2.1.2. Ferruginol

**Figure 2:**
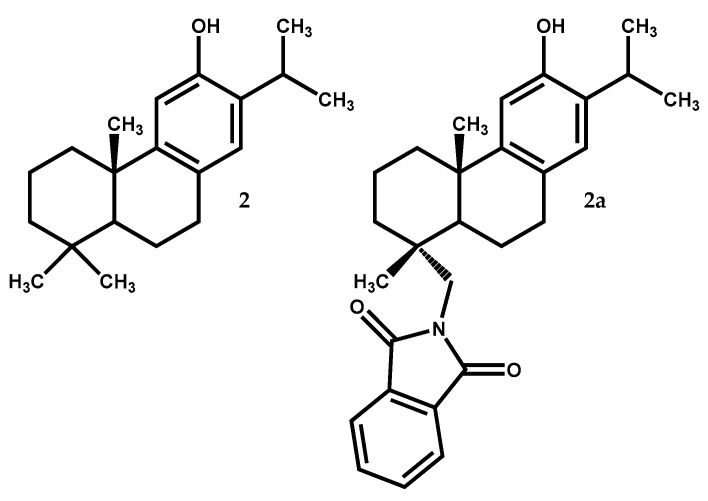
Ferruginol (**2**) from *Juniperus* species and the most active antiviral derivative (**2a**).

Ferruginol (2) (Figure 2) is, like dehydroabietic acid, a tricyclic diterpene with an aromatic ring but without the C-18 carboxylic acid and with a hydroxyl group at C-12. It is widely distributed in *Juniperus* genus [13,35], being particularly abundant in hexane extract of *J. excelsa* berries (32.9% of all the detected compounds) [36]. Several previous reports showed that this compound exhibits a great diversity of bioactivities such as anti-acaricide, antiplasmodial, nematicidal, antibacterial, antileishmanial, antiviral, antifungal, and antitumoral [26,36,37,38,39,40,41].

A recent study [42] showed that ferruginol (2) has antitumor activity, presenting inhibitory effects on HepG2 (IC_50_ = 11.4 ± 2.9 μg/mL, 39.8 μM) and Hep3B (IC_50_ = 19.4 ± 4.3 μg/mL, 67.7 μM) cell lines, without affecting the normal hepatocyte line L-02 viability (IC_50_ > 100 μg/mL, 349 μM). However, we would like to point out that the results previously mentioned have a standard deviation of around 20% of the value of the mean, which impairs the scientific impact of the results. Since ferruginol (2) exhibited the highest activity against Hep3B and HepG2 cell lines, the authors also assessed the mechanisms of apoptosis caused by compound (2). The results indicated that ferruginol (2) downregulated the expression levels of anti-apoptotic protein Bcl-2 (related to mitochondrial apoptosis pathway) and upregulated pro-apoptotic proteins Bcl-2-associated X (Bax), caspase-3, and caspase-9 [42].

Increased production, accumulation, and aggregation of the neurotoxic peptide amyloid-β (Aβ) within the brain triggers severe molecular changes affecting many signaling pathways associated with neuronal metabolism, signaling, and neuronal communication, leading to spatial memory loss and learning impairment associated with Alzheimer’s disease [43]. Amyloid β oligomers induce an imbalance in the calcium signaling kinases (vital for maintaining the integrity and functionality of synapses), which leads to progressive impairment of the synaptic connections, altering the capacity for hippocampal long-term potentiation (LTP) resulting in neuronal apoptosis [44]. A recent study by Zolezzi and colleagues [45] found that ferruginol (2) might have a potential neuroprotective role in neurodegenerative alterations. Their study reports that 10 μM of ferruginol induce an increase in calcium intracellular levels in hippocampal neurons from mice and promote neuroprotection against apoptosis, synaptic protein loss, and LTP inhibition triggered by amyloid β oligomers. The capacity of ferruginol to induce an increase in calcium was correlated with an increase in Ca^2+^/calmodulin-dependent protein kinase II (CaMKII) and in the active form of protein kinase C (PKC) in hippocampal slices, indicating that the changes in the LTP process and the calcium levels may be intermediated by the activation of calcium-dependent mechanisms involving PKC and CaMKII [46].

Furthermore, ferruginol (2) is the starting material for the synthesis of several compounds with high activity level and less secondary effects. In the work by Roa-Linares and colleagues [47], ferruginol and two analogues, showed relevant antiviral activity against Dengue Virus type 2, human Herpesvirus type 1, and human Herpesvirus type 2. The ferruginol derivative with a phthalimide moiety at C-18 (2a) (Figure 2), was ten times better (EC_50_ = 1.4 µM) than the reference ribavirin (EC_50_ = 13.5 µM) against Dengue Virus type 2 in a post-infection treatment and with a selectivity index value of 57.7, which indicates that this compound presents great potential as a therapeutic agent and should be aimed for further biopharmaceutical and pre-clinical studies [47].

#### 2.1.3. Hinokiol

**Figure 3:**
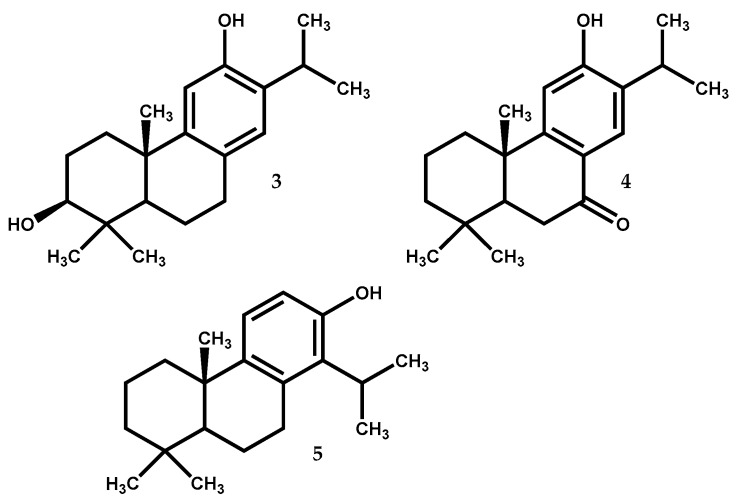
Diterpenes (**3–5**) from *Juniperus* species with significant pharmacological potential.

Hinokiol (3) (Figure 3) is a 3β,12-dihydroxy-abieta-8,11,13-trien present in *Juniperus* species, e.g., *J. brevifolia*, *J. chinensis*, *J. excelsa*, *J. phoenicea*, *Juniperus procera* Hochst. ex Endl. *Juniperus przewalskii* Kom. and *Juniperus squamata* Buch.-Ham. ex D.Don [12,13], with interest for the scientific community due to its pharmacological potential, since this compound has been reported to inhibit the generation of nitric oxide (NO) and TNF-α, as well as the production of pro-inflammatory enzymes from lipopolysaccharide-stimulated RAW macrophages [48,49]. Antioxidant [50] and hepatoprotective [51] effects have also been reported for hinokiol, as well as antitumor properties against human ovarian carcinoma (HO-8910) and cervical carcinoma (HeLa) cell lines [52].

In a more recent study, Wang and colleagues [53] reported the inhibition of voltage-gated Na^+^ channels (VGSCs) by hinokiol at 30 μM, in rat hippocampal CA1 neurons, differentiated NG108-15 cells and neuroblastoma N2A cells; VGSCs are crucial in the excitability of neurons since they permit the influx of Na^+^ during the upstroke phase of action potential, which ensures the quality of rapid signal transmission in the nervous system [53]. The VGSC inhibition by hinokiol presented in this work could be interpreted as an anti-anxiety, anaesthetic or anticonvulsant activity, but further research is necessary to clarify this topic and to hypothesize about future pharmacological applications.

#### 2.1.4. Sugiol

Sugiol (4) (Figure 3), 12-hydroxy-abieta-8,11,13-triene-7-one is widely distributed in the Cupressaceae family, being found in *J. brevifolia*, *J. chinensis*, *J. communis*, *Juniperus polycarpos* K.Koch, *J. procera*, *Juniperus rigida* var. *conferta* (Parl.) Patschke (syn. *J. conferta* Parl.) and *Juniperus formosana* Hayata [12,13,16,54,55]. Sugiol presents hepatoprotective [51] and antioxidant properties [56].

Bajpai and Kang [57] evaluated sugiol for tyrosinase and α-glucosidase inhibitory activity in vitro, in terms of its antimelanogenesis and antidiabetic potential, respectively. The results showed that sugiol at the concentration range of 0.100 to 10 mg/mL presented efficacy on inhibiting α-glucosidase (12.34 to 63.47% of inhibition) similar to acarbose (19.2 to 65.5% of inhibition at same concentration range), while at concentration 0.020 to 0.50 mg/mL, sugiol inhibits 28.2 to 67.4% of tyrosinase activity, only a little less active than kojic acid used as reference (32.4 to 76.5% inhibition at the same concentration range).

The Bajpai research group [58] reports also the potential of sugiol as antiviral once it inhibits the growth of H_1_N_1_ influenza virus in a cytopathogenic reduction assay using Madin-Darby canine kidney (MDCK) cell line. Severe cytopathic effect occurred in MDCK cells exposed to H_1_N_1_ influenza virus but in MDCK cells treated with sugiol (500 µg/mL) along with H_1_N_1_ influenza virus, cytopathic effect was absent. In fact, MDCK cells treated with sugiol showed similar morphology to control MDCK cells that were not exposed to H_1_N_1_ influenza virus.

Jung et al. [59] showed that sugiol may be useful against human solid tumors as an inhibitor of transketolase (TKT) and of the signal transducer and activator of transcription 3 (STAT3). In fact, the TKT reaction plays a crucial role in the pentose phosphate pathway, and its inhibition interrupts the production of FAD, NAD(P)^+^, CoA, and ATP, as well as the synthesis of DNA and RNA in cancer cells [60], while STAT3 inhibition plays an important role in the induction of cancer cells apoptosis [61]. In the work by Jung et al. [59], STAT3 activation was 40% inhibited by 20 μM of sugiol in DU145 prostate cancer cells, limiting their proliferation through cell cycle arrest at the G1/S checkpoint. The mechanism of inhibition proposed indicates that inhibition of TKT by sugiol imply ROS-mediated ERK activation and ERK activated phosphorylates STAT3 on Ser727 and recruits a protein tyrosine phosphatase MEG2, which dephosphorylates STAT3 on Tyr705 leading to the inhibition of STAT3 [59].

A very recent study [62] showed that sugiol reduced the cell viability of human pancreatic cancer cells (Mia-PaCa2) in a concentration-dependent manner being the IC_50_ value of 15 μM. The cytotoxic activity of sugiol was found to be caused by reactive oxygen species (ROS)-mediated alterations in mitochondrial membrane potential (MMP), in conjunction with an upregulation of Bax expression (an inducer of apoptosis) and a downregulation of Bcl-2 expression (an antiapoptotic protein). Additionally, the study indicates that sugiol also caused cell cycle arrest in G2/M phase of the cell cycle, ultimately leading to apoptosis. Furthermore, sugiol also inhibited the migratory capacity of Mia-PaCa2 cells at 15 μM concentration. This study suggests that sugiol is a very good candidate to in vivo evaluation against pancreatic cancer. Unfortunately, the authors of this study have not evaluated the compounds cytotoxicity towards a non-tumor cell line under the same conditions and did not use an approved clinical drug as positive control. If they had, they would have increased the impact of their work and its contribution to the field.

#### 2.1.5. Totarol

The compound totarol (5) (Figure 3) is a tricyclic phenolic diterpene with a totarane skeleton. It is found in several *Juniperus* species such as *J. brevifolia*, *J. chinensis*, *J. communis*, *J. conferta*, *J. excelsa*, *J. formosana*, *J. phoenicea*, *J. procera* and *Juniperus drupaceae* Labill. [12,13]. It is the most abundant compound in the hexane extract of *J. brevifolia* bark (11 mg of compound by 100 mg of extract) [54], being also found in species from other genus [63]. This compound seems to be a good bet towards new interesting active drugs development since it displays a range of interesting bioactivities such as antibacterial [64,65,66], antimycobacterial [38], antileishmanial [36], antimalarial [67,68], antistaphylococcal activity caused by efflux inhibitory properties [69], as well as nematicidal activity and antifouling attributes [36]. Furthermore, totarol could also be used as activity enhancer of some conventional drugs [70].

A promising antimicrobial target is the bacterial cell division machinery and totarol (5), by perturbing the cell division, has the capacity to restrain bacterial growth [71]. A recent study [72] focused on the molecular targets and mechanism of action of totarol in *Bacillus subtilis*. Their quantitative proteome analysis showed that diterpene (5) induced changes in 139 proteins expression levels. The same study also reports that *Bacillus subtilis* major central metabolic dehydrogenases are repressed by totarol (5) at IC_50_ = 1.5 μM leading to metabolic shutdown in the bacteria.

Another study [73] reports that totarol (5) has vascular protective effects in vivo, by activating the protein kinase B/heme oxygenase-1 (PKB/HO-1) pathway, further increasing superoxide dismutase (SOD) and antioxidant glutathione (GSH) levels, which leads to ischemia-induced brain injury suppression. An in vitro assay showed totarol as no toxicity on cerebellar granule cells (CGC) at various concentrations (1 to 5 μM), which strengthens its protective properties. In order to simulate the situation of patients with acute stroke, a post-ischemia administration of totarol in rats (1 and 10 μg/kg) was used. The results showed considerable decreases in infarct volume compared with the untreated group. Moreover, totarol treatment (1 and 10 μg/kg) radically enhanced the ischemia-induced neurological deficit. The study also reported notably infarct volume reduction with 10 μg/kg of totarol administration.

### 2.2. Flavonoids

#### 2.2.1. Amentoflavone

**Figure 4:**
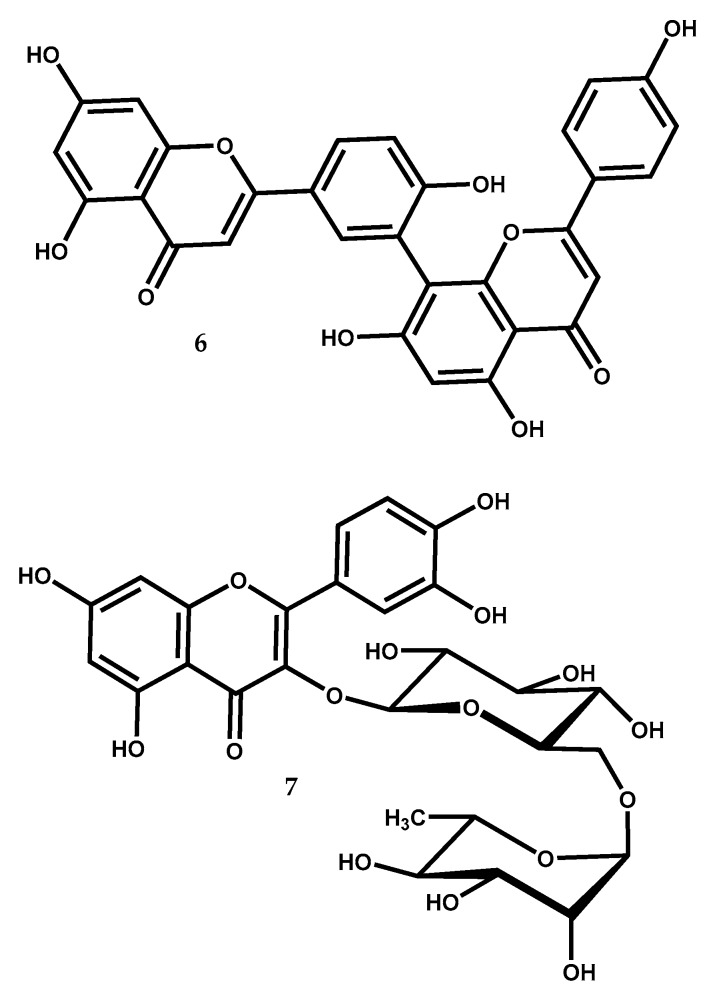
The bioactive flavonoids amentoflavone (**6**) and rutin (**7**) from *Juniperus* species.

Amentoflavone (6) (Figure 4), is a flavonoid dimer composed by two apigenin units linked by a carbon-carbon bond between C-8 and C-3′, belonging to the biflavonoid family of compounds and is found in several *Juniperus* species, like: *J. oxycedrus*, *J. phoenicea*, *J. rigida*, *J. virginiana*, *J. chinensis*, *J. communis*, *J. drupacea*, *J. foetidissima*, *Juniperus bermudiana* L., *Juniperus indica* Bertol., *Juniperus macrocarpa* Sm., and *Juniperus occidentalis* Hook. [12,13,74].

Amentoflavone (6) possesses a wide variety of bioactivities, such as antiphotoaging [75], antifungal [76], antimicrobial [77], antioxidant [78], anti-inflammatory [79], antidiabetic [80], antipsoriasis [81], diuretic [82] and antitumor [83,84], as well as neuroprotective [85] and osteogenesis effects [86], and it confers cardiovascular injury protection [87]. Although all these bioactivities are well reviewed with great detail by Yu et al. [74] there are still some studies that are worth mentioning that were not included in the review.

Inhibition of prostaglandin D2 (PGD2) has been found as a pharmacological mechanism for the treatment of androgenic alopecia (i.e., pattern hair loss) [88]. A study [89] found that amentoflavone (6) might inhibit PGD2 synthesis and that it has acceptable skin permeability as well as not being irritating or corrosive to skin, suggesting that amentoflavone (6) can be used to develop safe and high-efficacy hair loss treatment.

Estrogens have a crucial role in the initiation and the progression of breast cancers. Aromatase catalyses the rate-limiting step in endogen/estrogen synthesis and its activity is stated to be higher in breast cancer [90]. Tascioglu et al. [91] showed that amentoflavone (6) could act as an aromatase inhibitor being determined the IC_50_ value as 93.6 μM in an in vitro assay.

In a very interesting study [92], the protective effect of amentoflavone (6) against Freund’s adjuvant induced arthritis in rats was evaluated. The findings show that treatment with 20 mg/kg and 40 mg/kg doses of amentoflavone (6) has suitable anti-arthritic properties since it demonstrates to positively control inflammation in the adjuvant induced arthritic rat model. Protective effects were also reported in another study [93], where it was demonstrated that amentoflavone (6) protected dopaminergic neurons against MPTP/MPP^+^-induced neurotoxicity. This neuroprotective activity may have its clinical application in the treatment of some central nervous system (CNS) diseases, such as Parkinson’s disease and ischemia.

Another study [94] examined the effects of amentoflavone in human ovarian cancer cell lines OVCAR-3 and SK-OV-3. The results showed that this biflavonoid (6) could considerably suppress cell propagation, block cell cycle progression at the G1/G0 phase and induce cell apoptosis. In both cell lines, amentoflavone (6) displayed dose- and time-dependent inhibition. In SK-OV-3 cells assay, after 48 h of treatment with compound (6) at 20 and 50 μM the cell viability decrease 15% and 20%, respectively, while with incubation time extended to 72 h, the decrease cell viability was 19% and 31% for the respective doses. In OVCAR-3 cells, the results were similar. Also, apoptotic cell population increased after 48 h and 72 h treatment at 20 μM and 50 μM. Furthermore, the results showed that amentoflavone (6) repressed the expression of S-phase kinase protein 2 (Skp2) through ROS/AMPK/mTOR signaling [94], which contributed to amentoflavone antitumor effect against ovarian cancer.

The amentoflavone (6) inhibitory activity on human aldo-keto reductase family 1 member B10 (AKR1B10), which is a detoxification enzyme involved in drug resistance, was studied [95]. The results showed that compound (6) decrease the growth of A549 human lung cancer cells in vitro and in vivo by potently inhibition of human AKR1B10 activity (IC_50_ = 1.54 μM).

A key transcription factor that responds to oxidative stress is nuclear factor erythroid 2-related factor 2 (Nrf2) and its activation is related with prevention of aging, inflammation and cancer [96]. A recent study [97] found that amentoflavone (6) could trigger Nrf2 activation through ROS-mediated activation of the p38-AKT/PKB pathway in HaCaT keratinocytes.

A dipeptidyl peptidase IV (DPP-IV) inhibitors increase the activation of glucagon-like peptide 1 (GLP-1) and glucose-dependent insulinotropic polypeptide (GIP), leading to the inhibition of secretion of glucagon and enhancement of β-cells functionality [98]. Thus the control of DPP-IV activity is an essential factor in management of type 2 diabetes, and amentoflavone (6), with an IC_50_ value of 3.9 ± 0.5 μM, was recognized as a potential DPP-IV inhibitor [99].

#### 2.2.2. Rutin

Rutin, 3,3′,4′,5,7-pentahydroxyflavone-3-rhamnoglucoside, and also known as quercetin 3-rutinoside (7) (Figure 4), is a flavonol found in many plants including *Juniperus* species like *J. communis*, *J. excelsa*, *J. foetidissima*, and *J. oxycedrus* [12,100].

Recently, two papers [101,102] have exhaustively reviewed the rutin (7) bioactivities and pharmacological potential. They showed that it possesses multiple pharmacological activities, including antioxidant, hepatoprotective, vasoprotective, anticarcinogenic, neuroprotective, cardioprotective and antidiabetic activities [101,102].

A recent work, not included in the mentioned reviews, reported that rutin can provide cardioprotective effect [103]. In this work, rutin at 50 μM was more effective than the cardioprotective agent dexrazoxane (DZR) at same concentration, in preventing pirarubicin-induced toxicity in rat cardiomyoblasts H9c2. The apoptosis rate of rutin (7) treatment after cells exposed to pirarubicin was nearly 20%, while DZR treatment reported an apoptosis rate of about 30% [103]. The authors propose that the protective effect of rutin (7) is related with its ability to scavenge intracellular ROS and inhibit cell apoptosis by modulating the transforming growth factor (TGF)-β1-p38 MAPK signaling pathway.

An interesting work by Parashar et al. [104] found that rutin (7) (100 mg/kg) could alleviate chronic unpredictable stress (CUS) in mice, acting as an antidepressant. Since CUS impairs locomotors abilities of animals [105], the fact that rutin (7) treated animals were more balanced and active than the untreated ones, indicates a strong stimulatory effect on balancing activity, muscle coordination and locomotion. In addition, animals treated with rutin (7) had intact memory and were capable to identify a previously encountered object, thus spending more time discovering a novel object [104]. Stressed animals treated with rutin (7) presented an intact hippocampus with morphology and cell number similar to control animals that were not subjected to CUS [104].

### 2.3. Lignans

#### Deoxypodophyllotoxin

**Figure 5:**
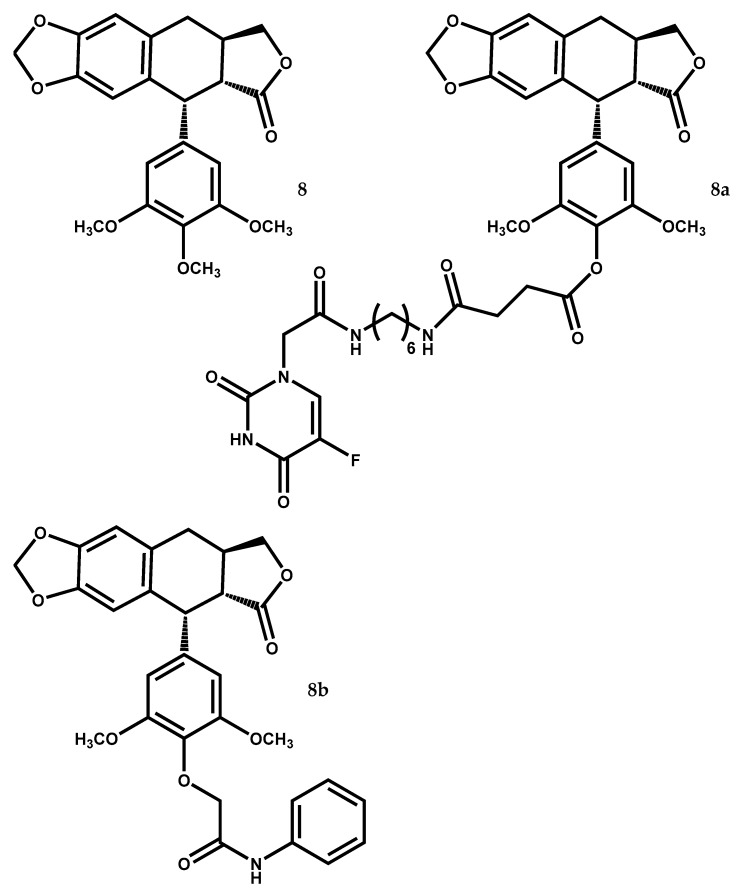
The bioactive lignan deoxypodophyllotoxin (**8**) identified in *Juniperus* species and its cytotoxic derivatives (**8a**) and (**8b**).

Deoxypodophyllotoxin (DPT) (8) (Figure 5) is an aryltetralin cyclolignan having been isolated from several *Juniperus* species like *J. virginiana*, *J. rigida*, *J. sabina*, *J. squamata*, *J. procera*, *J. bermudiana*, *J. chinensis*, *J. communis*, *J. phoenicea*, *Juniperus procumbens* (Siebold ex Endl.) Miq. *Juniperus recurva* Buch.-Ham. ex D.Don, *Juniperus taxifolia* Hook. & Arn. *Juniperus thurifera* L., and *Juniperus* x *media* V.D. Dmitriev [12,13,106,107].

The main characteristic of this compound is its great cytotoxic potential as reported in several studies [108,109,110,111,112]. Furthermore, DPT (8) also reported anti-inflammatory [113] and anti-angiogenic [109,111] properties.

A study [114] showed that deoxypodophyllotoxin (8) has a significant cytotoxic activity in vitro since it has inhibited the growth of numerous cancer cell lines (i.e., human glioblastoma-astrocytoma U-87 MG, human glioblastoma SF126, gastric carcinoma SGC-7901, gastric carcinoma BGC-823, ovarian carcinoma HO-8910, human ovarian carcinoma SK-0V-3, human colon carcinoma HT-29, breast carcinoma MDA-MB-231 and human choriocarcinoma JeG-3) with IC_50_ values varying from 13.95 to 26.72 nM, while the clinical anticancer drug etoposide was less efficient (IC_50_ ≥ 73.57 nM) [114]. Furthermore, the same study [114], also suggests that deoxypodophyllotoxin (8) treatment resulted in a dose- and time-dependent induction of apoptosis via caspase-dependent pathways by decreasing the expression of cyclin-dependent protein kinase 2 (Cdc2), cyclin B1, and cell division cycle 25C protein (Cdc25C), leading to cell cycle arrest in G2/M phase.

A recent study [115] also showed that DPT (8) at 5 nM induced G2/M cell cycle arrest in both human breast cancer cells MCF-7 (MCF-7/S) and their acquired resistant cells (MCF-7/A), while paclitaxel (10 nM) showed no effect on the cell cycle progression of the MCF-7/A cells. Besides that, DPT (8) exhibited antiproliferative activity against the MCF-7/S and MCF-7/A cell lines, with IC_50_ values of 10.61 ± 1.09 nM and 5.86 ± 0.30 nM respectively, with a resistance index (RI) [(IC_50_ of MCF-7/A cell line)/(IC_50_ of MCF-7/S cell line)] of 0.552 [115]. These values were better than the ones obtained by paclitaxel and etoposide [115]. Furthermore, DPT (8) at 12.5 mg/kg, suppressed in vivo the tumor growth in MCF-7/S and in MCF-7/A xenograft mice, exhibiting tumor volume growth inhibition of 49.62% in the MCF-7/S xenografts, approaching the tumor volume growth inhibition of paclitaxel (53.86% at 12.5 mg/kg). In addition, DPT (8) has potential to be a new microtubule inhibitor for breast cancer treatment since its antitubulin polymerization activity showed the absence of the polymerized tubulin, indicating that deoxypodophyllotoxin disrupted microtubule assembly in a different manner than paclitaxel [115]. The results presented by Zang et al. [115] also confirmed that deoxypodophyllotoxin (8) was not a substrate of the P-gp efflux pump and could overcome P-gp-mediated multi-drug resistance, unlike what happens with paclitaxel which is a P-glycoprotein (P-gp) efflux pump substrate [116].

An in vivo study [117] showed the antitumor property of deoxypodophyllotoxin (8) on MDA-MB-231 human breast cancer xenografts in BALB/c nude mice in a concentration-dependent manner. Deoxypodophyllotoxin (8) was combined with hydroxypropyl-β-cyclodextrin (DPT-HP-β-CD) in order to turn it more soluble and facilitate its intravenous administration. The results revealed that DPT (8) exhibited strong inhibitory effect and great antitumor activity, being the treatment with DPT-HP-β-CD (20 mg/kg) in MDA-MB-231 xenograft more efficient than the ones with etoposide (20 mg/kg) and docetaxel (20 mg/kg) [117], two anticancer drugs in clinical therapeutic [118,119]. The authors [117] also point out that, similar to other cancer chemotherapy drugs, DPT-HP-β-CD treatment caused gastrointestinal reactions after intravenous injection, consequential reducing food intake, which led to weight loss in the mice.

An in vitro study from Hu et al. [120] investigated the cytotoxic effect of DPT (8) on human prostate cancer DU-145 cells and its potential action mechanism. The results revealed that DPT (8) induced cell apoptosis and inhibited cell proliferation. Detection of high levels of the caspase-3 expression suggests that caspase-mediated pathways were involved in DPT-induced apoptosis. Moreover, the authors suggest that apoptosis was also induced through downregulation of the levels of phosphorylated Akt and activation of the p53/B-cell lymphoma 2 associated X protein/phosphatase and tensin homolog (i.e., Akt/p53/Bax/PTEN) signaling pathway [120]. Although this work must be emphasized because it exposes a new target involved in the DPT (8) mechanism of action, no control was used nor were IC_50_ values against DU-145 cells line presented, which is a misfortune since it decreases the scientific impact of the study.

Parthanatos is a unique cell-death pathway that is distinctive from necrosis, apoptosis or other recognized forms of cell death. It is a process dependent on the over activation of the nuclear enzyme poly (ADP-ribose) polymerase 1 (PARP-1), causing it to synthesize a massive quantity of PAR polymer until reaching toxic levels, resulting in large-scale chromatin condensation and DNA fragmentation, leading to cell death [121]. A study [122] found that DPT (8) triggered parthanatos in rat C6, human SHG-44 and U87 glioma cell lines via induction of excessive reactive oxygen species (ROS). In addition, alterations of parthanatos-related proteins triggered by DPT (8) occurred in a dose and time dependent manner and involved the induced cytoplasmic accumulation of PAR polymer in SHG-44 and C6 glioma cells as well as the upregulation in the nuclear level of AIF and in the cytoplasmic and nuclear levels of PARP-1 [122].

ROS production plays a crucial role in apoptosis signaling, leading to cancer cell death [123], but it also can trigger autophagy [124]. Since autophagy is a degradation process in intracellular organelles that occurs when cells undergo nutrition deprivation and external stimulus, its activation is essential for preserving intracellular homeostasis and allowing the cell to survive [125]. An interesting study [126], demonstrated that deoxypodophyllotoxin (8) induces both autophagy and apoptosis in osteosarcoma U2OS cells, through modification of mitochondrial membrane potential (MMP), which is related with generation of ROS. Furthermore, DPT (8) suppressed the PI3 K/AKT/mTOR signaling cascades, a pathway that leads the autophagy activation. Hence, these results indicate that deoxypodophyllotoxin (8) triggers simultaneously cytoprotective autophagy and cytotoxic apoptosis.

DPT (8) was used in a recent study [127], to establish a physiologically based pharmacokinetic-pharmacodynamic (PBPK-PD) model that allowed to predict the tumor growth in human lung carcinoma NCI-H460 tumor-bearing mice during deoxypodophyllotoxin (8) multi-dose treatment, as well as in gastric cancer SGC-7901 tumor-bearing mice. Briefly, the PBPK-PD model uses in vitro/in vivo pharmacodynamic correlations and predicts antitumor effectiveness in tumor-bearing mice based on in vitro pharmacodynamics assays results. The authors defend that this PBPK-PD model could be use with other compounds besides DPT, permitting a faster dose regimen design and anticancer candidate screening in drug discovery processes.

Derivative compounds from deoxypodophyllotoxin (8) also present great potential as anticancer drugs, as it is shown in a study from Guan et al. [128]. In their study, cytotoxic activity of various deoxypodophyllotoxin–5-fluorouracil hybrid compounds were evaluated using four human cancer cell lines and the human lung fibroblast non-tumoral cell line WI-38. The majority of the hybrids were more potent in their cytotoxicity to the four tumor cell lines and presented reduced toxicity against the normal cell line than the reference compounds etoposide and 5-FU. The most promising compound was 4′-*O*-demethyl-4-deoxypodophyllotoxin-4′-yl 4-((6-(2-(5-fluorouracil-yl)acetamido) hexyl) amino)-4-oxobutanoate (8a) (Figure 5) that presented IC_50_ values of 0.27 to 4.03 μM against HeLa, A549, HCT-8 and HepG2 cells, being less toxic (IC_50_ = 113.8 μM) to WI-38 cells than 5-FU and etoposide (IC_50_ values of 78.52 μM and 35.8 μM respectively) [128]. Furthermore, this hybrid compound (8a) can inhibit A549 cell migration by up-regulation TIMP-1 and down-regulation matrix metallopeptidase 9 (MMP-9), as well as cause cell-cycle arrest in the G2/M phase by affecting levels of the cell-cycle regulators p-cdc2, cdc2 and cyclin B1 [128].

The same deoxypodophyllotoxin derivative (8a) (Figure 5), named C069 by Xiang et al. [129], could have antiproliferative effects in human umbilical vein endothelial cells (HUVEC), in a dose- and time-dependent way. C069 (8a) at concentrations of 0.1 and 0.3 μM, showed better antiproliferative activity than etoposide at 1 μM, and low cytotoxicity against human normal lung cells WI-38 [129]. Since HUVEC represent a model cell line used to study angiogenesis processes, its non-proliferation is translated as an anti-angiogenesis property of C069 (8a).

Zhu et al. [130], showed that other DPT derivative (8b) (Figure 5), exhibits the IC_50_ values of 0.22 ± 0.02 μM against MGC-803 cells, being more active than the reference etoposide (IC_50_ values > 10 μM against the same cells line) and it can cause cell cycle arrest in G2/M phase through regulation of cell cycle check point proteins expression, such as p21, cdc25c, CDK1, cyclin A, and cyclin B. The same derivative (8b) at 4 mg/kg was also able to reduce in 45.56% the weights and volumes of HepG2 xenografts in mice in just 14 days [130].

Despise the impressive clinical efficacy of the deoxypodophyllotoxin (8) and its derivatives, their therapeutic use still needs to overcome some difficulties like its poor water solubility [131] and rapid elimination [132].

## 3. Conclusions

In conclusion, *Juniperus* genus is very rich in species and promising metabolites with pharmaceutical potential, being *J. communis* and *J. oxycedrus* the two most studied species in terms of their phytochemistry, pharmacological and therapeutic effects.

As a summary, the effects of *Juniperus* secondary metabolites and the level of activity/mechanism of action are shown in Table 1.

Regarding bioactivities, antitumor activity is by far the most studied, being followed by antiviral and antibacterial activities, with several works researching compounds found on *Juniperus* species mainly for these properties.

From the compounds mentioned in this review, deoxypodophyllotoxin (8) appears to be the most promising one in terms of development into a pharmaceutical natural drug, since it has reported antitumor effects against breast cancer acquired resistant cells (MCF-7/A), with IC_50_ = 5.86 nM, a very interesting value in the nanomolar level. However, their therapeutic use still needs to overcome obstacles like its poor water solubility. A deoxypodophyllotoxin derivative more soluble could do the trick. The dehydroabietic acid derivative 1a also appears to be a good bet for further studies and development since it has shown IC_50_ values between 0.7–1.2 μM against PC-3, SK-OV-3, MCF-7 and MDA-MB-231 tumor cell lines, an activity higher than the one exhibited by the anticancer agent 5-FU used clinically, and with significant selectivity once dehydroabietic acid derivative 1a displayed very weak cytotoxicity against normal cells.

The majority of the studies addressed in this review were made at the in vitro scale, with only a handful being done in in vivo. In fact, this is only the first step of a long, expensive, and very selective route until it can be declared as a compound with real potential to be a new drug, that is, with therapeutic application or as a new head of series. Thus, while this review work outlines the most promising compounds on which more studies are published in recent years, we are convinced that only the two compounds highlighted in the previous paragraph will be interesting enough to attract the attention of the pharmaceutical industry.

In light of this, the more active and promising compounds presented in *Juniperus* species should be taken to the next step, with future works aiming to in vivo testing assessment of them, particularly the ones with antitumor effects.

On the other hand, studies regarding any bioactivity assay of any compound should always present IC_50_ values of a reference compound in order to increases their scientific impact and facilitate results comparison.

This review hopes to demonstrate the *Juniperus* species value and their importance as a source of metabolites with relevant pharmaceutical potential.

## Figures and Tables

**Table 1:** The key points of each secondary metabolite highlighted.

Compound	Biological Activity (Tested Model)	Level of Activity ^a^ (Control/Mechanism ^b^)	Ref.
**1a**	Antitumor (PC-3, SK-OV-3, MDA-MB-231 and MCF-7 cell lines)	IC_50_ = 0.7–1.2 μM (IC_50_ = 5.2–24.5 μM to 5-FU)	[33]
**1b**	Antibacterial (*Staphylococcus aureus* Newman)	MIC = 0.63–1.2 μM (MIC = 0.54–1.1 μM to vancomycin)	[34]
**2**	Antitumor (HepG2 cell line)	IC_50_ = 39.8 μM (low cytotoxicity to L-02 cell line)	[42]
	Neuroprotective (hippocampal neurons from mice)	At 10 μM cause ↑ calcium intracellular)	[45]
**2a**	Antiviral (Dengue Virus type 2)	EC_50_ = 1.4 µM with SI = 57.7 (EC_50_ = 13.5 µM to ribavirin)	[47]
**3**	Neurons excitability (rat hippocampal CA1 neurons)	At 30 µM cause inhibition of VGSC	[53]
**4**	Antidiabetic (α–glucosidase inhibition)	At 33.2 mM cause more than 65% of inhibition (at 15.4 mM acarbose cause identical inhibition)	[57]
	Antimelanogenesis (tyrosinase inhibition)	At 1.7 mM cause more than 65% of inhibition (at 3.5 mM kojic acid cause identical inhibition)	[57]
	Antiviral (MDCK cell line exposed to H_1_N_1_ virus)	At 1.7 mM protect against severe cytopathic effect caused by H_1_N_1_ virus	[58]
	Antitumor (DU145 cell line)	At 20 μM the STAT3 activation was 40% inhibited	[59]
	Antitumor (Mia-PaCa2 cell line)	IC_50_ = 15 μM (↑ Bax expression, ↑ ROS–mediated alterations, ↓ Bcl–2 expression, ↓ migratory capacity	[62]
**5**	Antibacterial (*Bacillus subtilis*)	At IC_50_ = 1.5 μM inhibition of metabolic dehydrogenases	[72]
	Vascular-protection (rats)	At 1–10 μg/kg ↓ infarct volume, ↑ ischemia–induced neurological deficit by activation of PKB/HO–1, SOD and GSH	[73]
**6**	Antitumor (aromatase inhibition)	IC_50_ = 93.6 μM	[91]
	Antitumor (OVCAR-3 and SK-OV-3)	20–50 μM cause ↓ cell propagation, block cell cycle progression at the G1/G0 phase and induce cell apoptosis	[94]
	Antitumor (A549)	IC_50_ = 1.54 μM (inhibition of human AKR1B10 activity)	[95]
	Anti-arthritis (adjuvant induced arthritic rats)	At 20–40 mg/kg cause ↓ inflammation	[92]
	Antidiabetic (DPP-IV inhibition)	IC_50_ = 3.9 μM	[99]
**7**	Cardioprotective (rat cardiomyoblasts H9c2)	At 50 μM exhibits an apoptosis rate of 20% after pirarubicin–induced toxicity (30% to dexrazoxane)	[103]
	Antidepressant (in mice)	At 100 mg/kg alleviate CUS	[104]
**8**	Antitumor (U-87 MG, SF126, SGC-7901, BGC-823, HO-8910, SK-0V-3, HT-29, MDA-MB-231, JeG-3)	IC_50_ = 13.95–26.72 nM by ↓ Cdc2 expression, ↓ cyclin B1, ↓ Cdc25C (IC_50_ ≥ 73.57 nM to etoposide)	[114]
	Antitumor (MCF-7/S, MCF-7/A)	IC_50_ = 5.86 nM, RI = 0.552 (paclitaxel and etoposide exhibit higher IC_50_ and RI)	[115]
	Antitumor (MCF-7/S and in MCF-7/A xenograft mice)	At 12.5 mg/kg 49.2% of tumour volume growth inhibition (identical to paclitaxel)	[115]
**8a**	Antitumor (HeLa, A549, HCT-8 and HepG2 cell lines)	IC_50_ = 0.27–4.03 μM, cell migration inhibition, ↑ TIMP-1 expression, ↓ MMP-9 expression, more selectivity than 5-Fu and etoposide	[128]
	Antitumor (HUVEC cell line)	At 0.1–0.3 μM higher activity and selectivity index than etoposide at 1 μM	[129]
**8b**	Antitumor (MGC-803 cell line)	IC_50_ = 0.22 μM (IC_50_ values > 10 μM to etoposide)	[130]
	Antitumor (HepG2 xenografts in mice)	At 4 mg/kg ↓ in 45.56% the weights and volumes of tumor	[130]

^a^ The activity level presented as half maximal inhibitory concentration; ^b^ When available, data about activity level of the clinical drug used as positive control and/or action mechanism are given. ↑: increased level; ↓: decrease level.

## Abbreviation

5-Fu	Fluorouracil
A375	Human malignant melanoma
A549	Human lung carcinoma
AIF	Apoptosis inducing factor
AKR1B10	Aldo-keto reductase family 1 member B10
AKT/PKB	Protein kinase B
AMPK	Adenosine monophosphate -activated protein kinase
ATP	Adenosine triphosphate
Aβ	Amyloid-β
Bax	Bcl-2-associated X
BChE	Butyrylcholinesterase
BGC-823	Gastric carcinoma
C6	Rat glial tumor
CaMKII	Ca^2+^/calmodulin-dependent protein kinase II
Cdc2	Cyclin-dependent protein kinase 2
Cdc25C	Cell division cycle 25C protein
CGC	Cerebellar granule cells
CNS	Central nervous system
CoA	Coenzyme A
CUS	Chronic unpredictable stress
DNA	Deoxyribonucleic acid
DNCB	2,4-Dinitrochlorobenzene
DPP-IV	Dipeptidyl peptidase IV
DPT	Deoxypodophyllotoxin
DPT-HP-β-CD	Mixture of deoxypodophyllotoxin with hydroxypropyl-β-cyclodextrin
DU145	Human prostate cancer
DZR	Dexrazoxane
EC_50_	Half maximal effective concentration
EJ138	Human bladder carcinoma
ERK	Extracellular signal-regulated kinase
FAD	Flavin adenine dinucleotide
GIP	Glucose-dependent insulinotropic polypeptide
GLP-1	Glucagon-like peptide 1
GSH	Glutathione
H9c2	Rat cardiomyoblasts
HaCaT	Nontumorigenic human epidermal cells
HCT-8	Human colorectal adenocarcinoma
HCT116	Human colon cancer
HeLa	Human cervical carcinoma
Hep3B	Human hepatoma
HepG2	Human hepatocellular carcinoma
HFF-1	Human normal fibroblast
HL-7702	Human liver normal
HO-8910	Human ovarian carcinoma
HT-29	Human colon carcinoma
HUVEC	Human umbilical vein endothelial cells
IC_50_	Half maximal inhibitory concentration
IgE	Immunoglobulin E
IL-4	Interleukin 4
JeG-3	Human choriocarcinoma
L-02	Human fetal hepatocyte normal cell line
LTP	Long-term potentiation
MCF-7/A	Acquired resistant human breast cancer
MCF-7/S	Human breast cancer
MCF-7	Human breast cancer
MDA-MB-231	Human breast adenocarcinoma
MDCK	Madin-Darby canine kidney cells
MGC-803	Human gastric cancer
Mia-PaCa2	Human pancreatic carcinoma
MIC	Minimum inhibitory concentration
MMP	Mitochondrial membrane potential
MMP-9	Matrix metallopeptidase 9
MPP^+^	1-methyl-4-phenylpyridinium
MPTP	1-methyl-4-phenyl-1,2,3,6-tetrahydropyridine
mTOR	Mammalian target of rapamycin
N2A	Mouse neuroblastoma
NAD(P)^+^	Nicotinamide adenine dinucleotide phosphate
NCI-H460	Human lung carcinoma
NO	Nitric oxide
Nrf2	Nuclear factor erythroid 2-related factor 2
OVCAR-3	Human ovarian adenocarcinoma
PAR	Poly (ADP-ribose)
PARP-1	Poly (ADP-ribose) synthetase 1
PBPK-PD	Physiologically based pharmacokinetic-pharmacodynamic
PC-3	Human prostate cancer
PGD2	Prostaglandin D2
PKB/HO-1	Protein kinase B/heme oxygenase-1
PKC	Protein kinase C
PTEN	Phosphatase and tensin homolog
RAW 267.4	Macrophage normal cell line
RNA	Ribonucleic acid
ROS	Reactive oxygen species
SF126	Human glioblastoma
SGC-7901	Gastric carcinoma
SHG-44	Human malignant glioma
SI	Selective index
SK-OV-3	Ovarian cancer
Skp2	S-phase kinase protein 2
SOD	Superoxide dismutase
STAT3	Signal transducer and activator of transcription 3
TGF	Transforming growth factor
TIMP-1	TIMP metallopeptidase inhibitor 1
TKT	Transketolase
TNF-α	Tumor necrosis factor α
U-87 MG	Human glioblastoma-astrocytoma
VGSC	Voltage-gated Na^+^ channels
WI-38	Human lung fibroblast normal cells
